# Endoscopic full-thickness resection versus endoscopic submucosal dissection in the treatment of colonic neoplastic lesions ≤ 30 mm—a single-center experience

**DOI:** 10.1007/s00464-021-08492-0

**Published:** 2021-04-15

**Authors:** Přemysl Falt, Jana Zapletalová, Ondřej Urban

**Affiliations:** 1 University Hospital and Faculty of Medicine, 2nd Department of Internal Medicine, Gastroenterology and Geriatrics, Palacký University, Olomouc, Czech Republic; 2 Department of Medical Biophysics, Faculty of Medicine, Palacký University, Olomouc, Czech Republic

**Keywords:** Endoscopic full-thickness resection, Endoscopic submucosal dissection, Colorectal cancer

## Abstract

Endoscopic full-thickness resection (FTR) is a novel technique of endoscopic treatment of colorectal neoplastic lesions not suitable for endoscopic polypectomy or mucosal resection. FTR appears to be a reasonable alternative to technically demanding endoscopic submucosal dissection (ESD) for lesions ≤ 30 mm. However, comparison between FTR and ESD has not been published yet and their mutual positioning in the treatment algorithm is still unclear. The purpose of the analysis was to evaluate efficacy and safety of FTR in the treatment of colorectal lesions ≤ 30 mm by comparing prospectively followed FTR cohort to retrospective ESD cohort in the setting of single tertiary endoscopy center. Primary outcomes were technical success rate, R0 resection and curative resection rate, and complication rate. A total of 52 patients in FTR and 50 patients in ESD group were treated between 2015 and 2018. Technical success rate was significantly higher in FTR group (92 vs. 74%, *P* = 0.01) as well as R0 resection rate (85 vs. 62%, *P* = 0.01) and curative resection rate (75 vs. 56%, *P* = 0.01). Complications occurred more frequently in ESD group (40 vs. 13%, *P* = 0.002), mainly due to high incidence of electrocoagulation syndrome (24 vs. 0%). Total procedure time was substantially shorter in FTR group (26.4 ± 11.0 min vs. estimated 90–240 min). Local residual neoplastic lesions were detected numerically more often in FTR group (12 vs. 5%, *P* = 0.12). No patient died during follow-up. Compared to ESD, FTR proved significantly higher technical success rate, higher R0 and curative resection rate, and shorter procedure time. In the FTR group, there were significantly less complications but higher incidence of local residual neoplasia. Further research including randomized trials is needed to compare both resection techniques.

## Introduction

Endoscopic full-thickness resection (FTR) is a novel technique for transmural resection of the colorectal neoplastic lesions using combination of over-the-scope clip application and cap-assisted snare resection [1]. It has been proven effective in the treatment of neoplastic lesions with non-lifting during submucosal injection, suspected early cancer lesions, local residual neoplasia lesions, and small subepithelial tumors [2–9]. Major limitation of FTR is limited extent of resection; therefore, only flat lesions ≤ 30 mm may be successfully treated. Alternatively, these lesions may be treated by endoscopic submucosal dissection (ESD). Colorectal ESD has been standardly used in some western endoscopy centers despite being considered technically demanding, time consuming, and risky, especially outside the rectum [10–13]. So far, there is no consensus on using FTR or ESD for colorectal neoplastic lesions potentially curable by both methods. As far as we know, comparison between FTR and ESD has not been published yet.

Our study was designed to evaluate the efficacy and safety of FTR compared to ESD in the treatment of colorectal neoplastic lesions ≤ 30 mm in the setting of single tertiary endoscopy center.

## Methods

### Setting

This study was conducted in a tertiary endoscopy center at Vítkovice Hospital in Ostrava and then at University Hospital in Olomouc, Czech Republic. Prospective part of the protocol was approved by the local ethics committee. All patients provided written informed consent. All procedures were performed by two experienced therapeutic endoscopists (P.F., O.U.) certified for both FTR and ESD.

### Patients

In FTR cohort, all consecutive patients treated by FTR for colorectal neoplastic lesions between August 2016 and September 2018 were included and prospectively followed. ESD cohort consists of all retrospective cases of ESD performed for colorectal neoplastic lesions between March 2015 and June 2018. All lesions with diameter > 30 mm, with bulky polypoid mass or lesions in the distal rectum were excluded from this analysis. Indication criteria in both groups were the same, and generally, there were patients with colorectal neoplastic lesions not suitable for endoscopic mucosal resection (EMR) due to risk of submucosal invasion, positive non-lifting sign, previous unsuccessfully treated local residual neoplasia, or subepithelial lesions. Prediction of superficial submucosal invasion was based on endoscopic appearance of the lesion (type 0 − IIc or 0 − IIa + IIc according to Paris classification [14], type 2 according to NICE classification [15], pit pattern Vi according to Kudo classification [16], and/or negative non-lifting sign). Lesions with clear signs of deep submucosal invasion (type III according to Paris classification, type 3 according to NICE classification, and/or pit pattern Vn according to Kudo classification) were not treated endoscopically.

### Procedures

All patients were admitted and underwent standard split-dose preparation with polyethylene glycol or polyethylene glycol combined with ascorbic acid. During the procedure, midazolam and/or fentanyl were given intravenously at a maximal dose of 8 mg and 100 µg, respectively. Patients were placed in the left lateral or supine position according to location of the lesion.

FTR was performed using standard adult colonoscope (Olympus CF-H180 or CF-HQ190, Olympus, Hamburg, Germany), original FTRD set (Ovesco Endoscopy, Tübingen, Germany) and carbon dioxide (CO_2_) insufflation. The lesion was reached by the colonoscope and its margin marked by FTRD marking probe. Then, the colonoscope was withdrawn and introduced back to the lesion with FTRD set attached. Lesion was grasped and pulled into the cap by FTRD grasper, OTC clip was deployed, and lesion was resected by preloaded monofilamentous snare using pure cut mode 100–120 W (Olympus ESG-100 or ESG-300). In case of snare failure, lesion was resected by standard polypectomy snare just above the OTS clip line. Specimen was extracted and pinned on a cork board, measured, and formalin fixed. Finally, colonoscope was reintroduced to inspect the resection site. Immediately after FTR, single intravenous shot of antibiotic (1000/200 mg of amoxicillin-clavulanate or 400 mg of ciprofloxacin) was administered.

ESD was performed using standard adult or pediatric colonoscope (Olympus CF-H180, CF-HQ190, PCF-H190DL or PCF-HQ190TL) with cap attached (Olympus D-201-14304) and CO_2_ insufflation. For submucosal injection, solution of 10% hydroxyethyl starch, epinephrine 1:50–100.000, and methylene blue was used. Initial circular incision and subsequent submucosal dissection were performed by DualKnife (Olympus KD-650U) or DualKnife J (KD-655U) using electrosurgical unit (Olympus ESG-100 or ESG-300) in PulseCut mode 40–60 W and ForcedCoag 10–60 W, respectively. Intraprocedural bleeding was treated by closed ESD knife, coagulation forceps (Olympus Coagrasper FD-411UR), or endoclips (Olympus EZ Clip HX-110UR or QuickClip2 HX-201YR-135). In some cases, endoscopist decided to complete ESD by snare resection using standard polypectomy snares (S-ESD, “simplified ESD”). Specimen was extracted and pinned on a cork board, measured, and formalin fixed.

### Outcomes

Primary outcome measures were technical success rate, R0 resection rate, curative resection rate, and complication rate. Technical success of FTR was defined as endoscopically complete *en bloc* resection achieved by the FTRD set, or by standard polypectomy snare after deployment of OTS clip and failure of preloaded snare. ESD was considered successful, if endoscopically complete *en bloc* resection is achieved by standard ESD or S-ESD using polypectomy snare after partial submucosal dissection. R0 resection indicates an *en bloc* and concurrently microscopically margin-negative (both lateral and vertical) resection. Curative resection was defined as a R0 resection without histological risk factors for lymphatic spread (deep submucosal invasion > 1000 µm, poor differentiation, lymphatic, vascular and perineural invasion, budding). Complication was defined as a condition changing standard process after the procedure and leading to surgery, prolonged length of stay, repeated admission, repeated colonoscopy, administration of blood transfusion, or antibiotics.

Secondary outcome was the occurrence of local residual neoplasia defined as endoscopic or microscopic presence of neoplastic tissue in the post-resection site during follow-up colonoscopy. Then, size of the specimen measured by pathologist, level of used sedation, total procedure time, length of hospital stay, and C-reactive protein level during 24 h after the procedure were reported.

### Statistical analysis

Quantitative parameters were compared using the Student´s paired t-test in case of normal distribution and the Mann–Whitney *U*-test in case of abnormal distribution. The Chi-squared test and the Fisher exact test were used for comparing qualitative parameters. Normality was verified using the Shapiro–Wilk normality test. *P* value < 0.05 was considered statistically significant. Analysis was calculated using software IBM SPSS Statistics version 22.

## Results

During a period between August 2016 and September 2018, a total of 54 patients were treated by means of FTR. Two cases performed because of suspected Hirschprung´s disease were excluded. During a period between March 2015 and June 2018, a total of 90 patients were treated by ESD. 40 patients were excluded from this analysis due to the lesion size > 30 mm (*n* = 34), bulky polypoid portion (*n* = 4), or anorectal location (*n* = 2).

Baseline patient and lesion characteristics are described in Table 1. Age and sex distribution were comparable. However, there were significant differences in morphology, mean size, and location of the lesions. Local residual neoplastic lesions dominated in the FTR group (54%) while type 0 − IIa + IIc (LST-NGPD) lesions prevailed in the ESD group (56%). Size range was the same in both groups (8–30 mm), but mean size was significantly smaller in FTR group (15.6 ± 5.1 vs. 21.7 ± 6.5 mm). Lesions treated by FTR were evenly distributed throughout the colon and 57% of them were localized proximally to the lienal flexure. Majority of lesions treated by ESD were in the rectum (64%) and only 10% above the lienal flexure. Histological type of the lesions was almost identical in both groups including rate of submucosal invasive cancer (29 vs. 28%).

**Table 1:** Baseline patient and lesion characteristics (*n* = 102)

	FTR (*n* = 52)	ESD (*n* = 50)	*P* value
Male, *n* (%)	41 (79)	36 (72)	NS
Age, mean ± SD, years	69.1 ± 10.3	66.3 ± 10.5	NS
Lesion Morphology, *n* (%)*			
0 – Is	1 (2)	6 (12)	
0 – IIa	7 (13)	–	
0 – IIc	7 (13)	1 (2)	< 0.0001
0 – IIa + Is (LST-GM)	1 (2)	9 (18)	
0 – IIa + IIc (LST-NGPD)	6 (12)	28 (56)	
LRN	28 (54)	4 (8)	
SET	2 (4)	2 (4)	
Lesion size, mean ± SD	15.6 ± 5.1	21.7 ± 6.5	< 0.0001
(range), mm	(8–30)	(8–30)	
Lesion location, *n* (%)			
Rectum	7 (13)	32 (64)	
Sigmoid	13 (25)	9 (18)	
Descending colon	2 (4)	4 (8)	< 0.0001
Transverse colon	8 (15)	3 (6)	
Ascending colon	10 (19)	–	
Cecum	7 (13)	2 (4)	
Periappendicular	5 (10)	–	
Histology, *n* (%)			
LGD adenoma	7 (13)	8 (16)	
HGD adenoma	20 (38)	21 (42)	
Intramucosal carcinoma	3 (6)	5 (10)	
Carcinoma sm^1^	10 (19)	7 (14)	NS
Carcinoma sm^2−3^	5 (10)	7 (14)	
NET	1 (2)	2 (4)	
GIST	1 (2)	–	
Scar without neoplasia	5 (10)	–	

*according to Paris classification [14]

*FTR* Full-Thickness Resection, *ESD* Endoscopic Submucosal Dissection, *LST-GM* Laterally Spreading Tumor-Granular Mixed, *LST-NGPD* Laterally Spreading Tumor-Non-Granular Pseudo-Depressed, *SD* Standard Deviation, *LRN* Local Residual Neoplasia, *SET* SubEpithelial Tumor, *LGD* Low-Grade Dysplasia, *HGD* High-Grade Dysplasia, *NET* NeuroEndocrine Tumor, *GIST* GastroIntestinal Stromal Tumor, *NS* Not Significant

Characteristics of the endoscopic treatment are stated in Table 2. Resection was significantly more often considered technically successful in FTR group (92 vs. 74%, *P* = 0.01). Failure of the technique was reported in only 4 FTR cases. In 2 of them, there was not possible to draw the lesion into the cap and in other 2 cases, preloaded snare got stuck and lesions had to be piecemeal resected by polypectomy snare. In ESD group, 13 cases were considered technical failure. Lesion was resected in piecemeal manner in 7, ESD was converted to EMR in 5, and complete resection was not possible in 1 case.

**Table 2:** Characteristics of the treatment (*n* = 102)

	FTR (*n* = 52)	ESD (*n* = 50)	*P*	Power of a test (1–β)
Technical success rate, *n* (%)	48 (92)	37 (74)	0.01	0.68
R0 resection, *n* (%)	44 (85)	31 (62)	0.01	0.76
Curative resection, *n* (%)	39 (75)	28 (56)	0.01	0.53
Subsequent surgical resection	5 (10)	6 (12)	NS	
Specimen size, mean ± SD (range), mm	22.1 ± 6.0 (6–34)	25.2 ± 7.0 (12–36)	0.03	
Transmural resection, *n* (%)	38/48 (79)	–	N/A	
Sedation, *n* (%)				
Unsedated	6 (12)	2 (4)		
Minimal sedation	17 (33)	19 (38)		
Moderate sedation	7 (13)	27 (54)	< 0.0001	
Deep sedation	17 (33)	2 (4)		
General anesthesia	5 (10)	–		
Procedure time, mean ± SD (range), min	26.4 ± 11.0 (16–65)	(> 90) (90–240)	N/A	
Length of stay, mean ± SD (range), days	1.7 ± 1.2 (0–7)	3.2 ± 1.9 (1–10)	< 0.0001	
CRP level, *n* (%)				
≥ 10 mg/l (in 5–9 h)	5/31 (16)	8/49 (16)	NS	
≥ 10 mg/l (in 18–22 h)	6/31 (19)	40/49 (82)	< 0.0001	
≥ 30 mg/l (in 18–22 h)	2/31 (6)	20/49 (41)	0.001	
Complications, *n* (%)	7 (13)	20 (40)	0.01	0.88
Perforation	1 (2)	4 (8)	NS	
Delayed bleeding	4 (8)	4 (8)	NS	
Coagulation syndrome	–	12 (24)	0,001	
Acute appendicitis	2 (4)	–	N/A	
Follow-up colonoscopy, *n* (%)	34/45 (76)	21/44 (48)	N/A	
LRN, *n* (%)	4/34 (12)	1/21 (5)	NS	0.12

*FTR* Full-Thickness Resection, *ESD* Endoscopic Submucosal Dissection, *SD* Standard Deviation, *NS* Not Significant, *N/A* Not Applicable, *CRP* C-Reactive Protein, *LRN* Local Residual Neoplasia

Also R0 resection was achieved more frequently in FTR group (85 vs. 62%, *P* = 0.01). In 8 cases of failed R0 resection, there were 4 cases of already described technical failure and 4 cases of histologically positive lateral margins. Positive vertical margin did not occur in FTR group. In ESD group, there were 13 cases of already mentioned technical failure, 3 cases of positive lateral, and 3 cases of positive vertical margin.

Treatment was believed to be curative more frequently in FTR than in ESD group (75 vs. 56%, *P* = 0.01). Apart from technical failures and failed R0 resections in FTR group, there were 4 cases of deep submucosal invasive cancer and 1 case of T2 cancer. All of them were referred to a surgical resection. In ESD group, there were 3 cases of R0 resected but deep submucosal invasive cancer. All of them were referred to surgery.

Despite comparable size range of the specimens (6–34 vs. 12–36 mm), their mean size was significantly smaller in FTR group (22.1 ± 6.0 vs. 25.2 ± 7.0 mm). Histologically confirmed transmural resection occurred in 79% (38/48) of technically successful FTR.

Patients in FTR group underwent the procedure more frequently in deep sedation or under general anesthesia (43 vs. 4%). Mean total procedure time was 26.4 ± 11.0 min in FTR group. There were no data enabling accurate determination of procedure times in ESD group. Nevertheless, according to performing endoscopists and nurses, procedure scheduling, and endoscopic snapshots, all ESD procedures lasted > 90 min and estimated procedure time range was 90–240 minute.

Mean length of stay in hospital after the procedure was significantly shorter in the FTR group (1.7 ± 1.2 vs. 3.2 ± 1.9 days). C-reactive protein (CRP) level after the procedure was measured in 60% in FTR and in 98% of cases in ESD group. In 5–9 h following the procedures, the CRP level was ≥ 10 mg/l in 16% of cases in both groups. However, there were significantly less cases of increased CRP level in the FTR group in 18–22 h, both with level ≥ 10 mg/l (19 vs. 82%) and ≥ 30 mg/l (6 vs. 41%).

Complications were reported less frequently in patients in the FTR group (13 vs. 40%, *P* = 0.002). Perforation occurred in 1 case in the FTR and in 4 cases in the ESD group. In both groups, there were 4 cases of delayed bleeding. In ESD group, there were 20 cases of electrocoagulation syndrome while none in FTR group. Two cases of acute appendicitis following FTR of periappendicular lesions were treated conservatively. No patient died during this study and follow-up.

Results of follow-up colonoscopy were known in 76% in FTR and 48% of patients in ESD group, and patients undergoing surgical resection were excluded. Local residual neoplasia (LRN) was detected in 12% (4/34) after FTR and in 5% (1/21) after ESD (*P* = 0.12).

## Discussion

FTR and ESD are advanced techniques of endoscopic resection of early colorectal neoplastic lesions not suitable for standard polypectomy or endoscopic mucosal resection (EMR). Indications for FTR and ESD are basically similar, namely lesion suspected of superficial submucosal invasion, lesions with non-lifting caused by submucosal fibrosis, local residual neoplasia after previous endoscopic treatment, and periappendicular lesions. Lesions with clear signs of deep invasion should not be treated endoscopically with the exception of selected patients too risky for surgical resection [17].

Comparison between FTR and ESD has not been published yet. Their mutual positioning in the algorithm for treatment of early colorectal neoplastic lesions is still unclear and needs to be defined in order to achieve maximal effectivity and safety. Colorectal ESD is considered to be an established method with a lot of scientific evidence, especially from Asian countries. It offers high success rate of complete resection that is virtually not limited in horizontal direction. On the other hand, ESD is technically demanding and time consuming even for highly experienced endoscopists and is associated with significant risk of complications. ESD in the rectum is regarded easiest and safest while in the colon more difficult, risky and in some cases even impossible [18]. Specimens obtained by ESD contains only submucosal layer and therefore precise depth of submucosal invasion cannot be measured. FTR is a novel technique with modest published evidence almost exclusively from European countries. FTR does not require extensive training, procedure time is substantially shorter, and risk of associated complications may be lower [6, 9]. FTR may be safely used throughout the colon, although insertion of the FTR cap may be difficult in some patients. Full-thickness specimen provides exact staging of invasive cancer and FTR does not hinder from consequent surgical resection. Main drawback of FTR is limited extent of resection (≤ 30 mm). Overall cost of FTR and ESD procedures is comparable in the Czech Republic.

The aim of our study was to analyze prospectively followed group of 52 patients treated by FTR in comparison with retrospective group of 50 patients treated by ESD for colorectal neoplastic lesions ≤ 30 mm during a period between 2015 and 2018. Lesions > 30 mm, lesions with polypoid mass, and lesions in the distal rectum were excluded from analyzed ESD cohort because they were not potentially resectable by FTR.

Mean age and numerical superiority of males were comparable and correspond to distribution of colorectal neoplasia in our population [19]. Significant differences in indications, morphology, and location of the lesions arose from endoscopists´ preference and limitations of the methods. LRN lesions dominating in the FTR group are relatively frequent consequence of previous piecemeal EMR. FTR seems to be an effective alternative to ESD and surgical resection for the treatment of difficult LRN [20, 21]. ESD of LRN is considered difficult and associated with up to 15% of perforation risk [22]. Distally located 0 − IIa + IIc (LST-NGPD) type lesions prevailing in the ESD group represent traditional indication for colorectal ESD because of their accessibility, risk of invasive cancer, and size often close to the limit of FTR [18]. Significantly smaller mean lesion size in FTR group (15.6 ± 5.1 vs. 21.7 ± 6.5 mm) corresponds to already discussed limits of FTR. In ESD cohort, 40 lesions > 30 mm were not analyzed. It is worth mentioning that technical success rate in this group was only 40%, significantly lower than 74% in analyzed group of lesions ≤ 30 mm. Despite explained differences in lesion type, size, and location, there was similar proportion of histological types of the lesions, including rate of submucosal invasive (29 vs. 28%) and superficially submucosal invasive cancer (19 vs. 14%). These numbers are higher in large cohort of 11.260 colorectal lesions treated by ESD (15.7% of submucosal and 8% of superficially submucosal invasive cancer) [23] and the largest single-center FTR cohort of 181 lesions so far published (16 and 11.6%, respectively) [3] but they correspond to our pilot Czech study on colorectal lesions treated by ESD (14% of superficially submucosal invasive cancer) [24].

Primary outcomes were technical success rate, R0 resection rate, curative resection rate, and complication rate. Technical success rate was significantly higher in FTR group (92 vs. 74%, *P* = 0,01) and it is comparable to published evidence—in larger cohorts (> 50) ranged between 88 and 97% [2–5, 8, 9]. In 3 cases, preloaded snare failed and FTR was finished by standard polypectomy snare achieving R0 resection. In ESD group, there were 16 cases of S-ESD finished by polypectomy snare but R0 resection was reported in 7 of them only. R0 resection rate was also significantly higher while using FTR (85 vs. 62%, *P* = 0.01), which is in accordance with already published papers (77–91%) [2–5, 8, 9]. R1 resection in FTR group was caused by positive horizontal margin in 6 cases; vertical margin was always negative, even in deeply submucosal invasive and T2 cancer lesions. R1 resection in ESD group was reported in 3 cases of positive horizontal and 3 cases of positive vertical margins. According to higher technical success and R0 resection rate, proportion of curative resections was also higher in FTR group (75 vs. 56%, *P* = 0.01). 5 cases of R0 resection in FTR and 3 cases of R0 resection in ESD group were found to be non-curative, in 5 cases due to deep submucosal invasion, in 1 case due to T2 stage, and in 2 cases for low-grade differentiation and perineural invasion. Proportion of patients after non-curative resection indicated for subsequent surgery was similar (10 vs. 12%). Apart from one focus of adenoma in the scar after ESD, all surgical specimens were negative for neoplasia and lymph node involvement.

Far higher proportion of procedures in deep sedation or under general anesthesia in FTR group (43 vs. 4%) was not related to the procedure itself but to often painful insertion of FTR set to the resection site. Deep sedation or general anesthesia was used in 67% of patients with lesions above the lienal flexure but only in 18% of patients with lesions in the rectum. In 2 cases of lesion in the cecum and ascending colon, insertion of FTR set in sedation was not successful and had to be repeated under general anesthesia.

Procedure time is well-known drawback of colorectal ESD and concurrently potential strength of FTR. Mean total procedure time of FTR in our study was 26.4 ± 11.0 min and that was strikingly shorter than estimated procedure time range between 90 and 240 min in the ESD group. It corresponds to European colorectal ESD experience, and mean procedure time 127 min in German cohort of 182 colorectal ESD might be an example [11].

Complication rate was significantly lower in the FTR group (13 vs. 40%, *P* = 0.002) and it is comparable to published prospective FTR cohorts with range between 7 and 13% [2–5, 9]. The difference was caused mainly by 20 cases of electrocoagulation syndrome in ESD and none in FTR arm. Reliable closure of defect by OTS clip without weakening of the intestinal wall and use of cutting current may be explanation for rare occurrence of electrocoagulation syndrome after FTR [25]. This hypothesis might be supported by CRP levels after the procedures. Proportion of cases with increased CRP level in 18–22 h following the procedure was significantly higher in ESD group than in FTR group, both with level ≥ 10 mg/l (82 vs. 19%) and ≥ 30 mg/l (41 vs. 6%). In the FTR group, there was only one case of delayed perforation (2%), resulting in surgery on third day after standard FTR procedure. Similar cases of delayed events were reported in the literature [3, 26, 27]. There were 4 cases of perforations in the ESD group (8%), which is in accordance with published evidence [28]. Two perforations were detected during the procedure and two were delayed. Two patients were treated conservatively and two by means of surgery. In 4 cases in each arm (8%), significant bleeding occurred, which is again comparable to published experience [28]. In the FTR group, 2 cases resolved without intervention and 2 patients were indicated for repeated colonoscopy without need for endoscopic hemostasis or transfusion. In the ESD group, 3 patients underwent repeated colonoscopy with endoscopic hemostasis and transfusions were administered in one of them. Acute appendicitis was diagnosed in 2 out of 5 cases of FTR for periappendicular lesions, both were treated conservatively by antibiotics. In a recent multicenter cohort of 50 periappendicular lesions, acute appendicitis occurred in 14%, 4 cases were treated conservatively, and 3 cases underwent appendectomy [29].

After exclusion of surgical treated patients, follow-up colonoscopy was performed in 76% in the FTR and 48% in the ESD group. Local residual neoplasia (LRN) was detected in 12% (4/34) after FTR and in 5% (1/21) after ESD, difference was not significant most likely due to low number of subjects (*P* = 0.12). In the FTR group, there were three cases of LRN after R1 resection and one case after R0 resection of periappendicular lesion. Relatively common incidence of LRN associated with FTR is known from published cohorts. In German single-center study of 154 follow-up colonoscopies, LRN was detected in 12% (19/154) of patients, 8 cases resulted from R0 resections [3]. In German multicenter FTR registry of 1178 patients, endoscopic follow-up was available in 58% and LRN lesions were detected in 13.5% [9]. Follow-up colonoscopy is therefore highly recommendable after FTR, whereas R0 resection achieved by ESD rarely leads to LRN (< 1%) [30, 31].

We are aware of several limitations of our study. Firstly, randomized controlled design would be necessary to objectively compare both methods. Different morphology and location of lesions are surely related to preference of endoscopists and hypothetically, it may prevent from performing a true randomized trial. However, histological types including advanced neoplastic lesions were evenly distributed in both groups. Secondly, in contrast to FTR, ESD cohort was made retrospectively, but precise records contained all needed data for analysis. Thirdly, number of patients in the study could be higher. On the other hand, colorectal lesions suitable for FTR or ESD are not common even in tertiary endoscopy centers and our study represents their real occurrence in clinical practice.

## Conclusions

In conclusion, our study is the first direct comparison between FTR and ESD ever reported. In the treatment of early colorectal neoplastic lesions ≤ 30 mm, technical success rate, R0 resection rate, and curative resection rate were significantly higher and procedure time substantially shorter while using FTR than ESD. Complications occurred significantly more frequently in ESD group owing to high incidence of electrocoagulation syndrome and numerically higher incidence of perforation. Local residual lesions were detected more frequently during follow-up endoscopies in the FTR group. Further research including randomized trials is necessary to determine efficacy and safety of FTR comparing to ESD and other techniques of endoscopic resection.
